# Testing the H56 Vaccine Delivered in 4 Different Adjuvants as a BCG-Booster in a Non-Human Primate Model of Tuberculosis

**DOI:** 10.1371/journal.pone.0161217

**Published:** 2016-08-15

**Authors:** Rolf Billeskov, Esterlina V. Tan, Marjorie Cang, Rodolfo M. Abalos, Jasmin Burgos, Bo Vestergaard Pedersen, Dennis Christensen, Else Marie Agger, Peter Andersen

**Affiliations:** 1 Department of Infectious Disease Immunology, Statens Serum Institut, Copenhagen, Denmark; 2 Vaccine Branch, Center for Cancer Research, National Cancer Institute, National Institutes of Health, Bethesda, MD, United States of America; 3 Leonard Wood Memorial (LWM) Center for Leprosy Research, Cebu, Philippines; 4 Department of Epidemiology Research, Statens Serum Institut, Copenhagen, Denmark; Colorado State University, UNITED STATES

## Abstract

The search for new and improved tuberculosis (TB) vaccines has focused on IFN-γ both for selecting antigens and for evaluating vaccine delivery strategies. The essential role of IFN-γ in endogenous host protection is well established, but it is still uncertain whether this also holds true for vaccine protection. Here we evaluate the H56 fusion protein vaccine as a BCG booster in a non-human primate (NHP) model of TB that closely recapitulates human TB pathogenesis. To date, only a handful of novel adjuvants have been tested in the NHP model of TB, and therefore we administered H56 in 3 novel cationic liposome adjuvants of increasing immunogenicity (CAF01, CAF04, CAF05) and compared them to H56 in the IC31^®^ adjuvant previously reported to promote protection in this model. The individual clinical parameters monitored during infection (weight, ESR, X-ray) all correlated with survival, and boosting BCG with H56 in all adjuvants resulted in better survival rates compared to BCG alone. The adjuvants promoted IFN-γ-responses of increasing intensity as measured by ELISPOT in the peripheral blood, but the level of vaccine-specific IFN-γ production did not correlate with or predict disease outcome. This study’s main outcome underscores the importance of the choice of adjuvant for TB subunit vaccines, and secondly it highlights the need for better correlates of protection in preclinical models of TB.

## Introduction

In the past decade more than a dozen new anti-tuberculosis (TB) vaccines have entered phase 1 or phase 2 trials to evaluate safety and immunogenicity [1, 2]. Recently the modified vaccinia virus expressing Ag85A (MVA85A) was the first new TB vaccine in almost 50 years to undergo a clinical efficacy trial [3]. During both its pre-clinical and clinical development program, this vaccine demonstrated efficient boosting of BCG-induced Th1 cells as measured by IFN-γ ELISPOT. The phase 2b trials conducted in South Africa and Senegal unfortunately showed that boosting BCG-vaccinated infants and HIV-1^+^ adults with MVA85A did not improve protection against TB compared to BCG only [3, 4]. The lack of protection in those trials underscores the importance of assessing other vaccine platforms and immunogens in the development of a new TB vaccine.

Recent data has suggested that vaccine-induced IFN-γ does not correlate with protection in mice [5–7] and BCG-immunized infants [8]. Other studies suggest a correlation between IFN-γ and protection in mice and cattle [9–11]. Verreck and colleagues found MVA85A-induced IFN-γ to correlate with protection in NHPs in terms of early pathology, but long-term survival was not done [12]. Hence, the essential role of endogenous IFN-γ has been extensively documented in protective anti-TB immunity [13, 14], but whether vaccine-induced IFN-γ correlates with long-term protection and survival in a NHP model of TB is uncertain.

Our group has focused on developing novel TB vaccine fusion proteins and suitable adjuvants. The H56 fusion protein (Ag85B-ESAT6-Rv2660c) is a vaccine developed to boost BCG in individuals with and without an existing TB infection [15, 16] and is currently undergoing clinical phase 2 testing. We have previously demonstrated that boosting BCG-immunized cynomolgus macaques with H56 in the T cell-inducing IC31^®^ adjuvant delays and reduces clinical disease upon infection with *Mycobacterium tuberculosis* (Mtb) and prevents anti-TNF triggered reactivation of latent infection [17]. As only a few different adjuvants have been tested in TB-related NHP research, we tested the importance of adjuvants and the magnitude of the vaccine-induced Th1 response measured as specific IFN-γ production in peripheral blood by comparing H56 in three liposomal adjuvants in development, Cationic Adjuvant Formulation 01 (CAF01 consisting of DDA/TDB), CAF04 (DDA/MMG), and CAF05 (DDA/TDB/poly(I:C)) [18–22]. These adjuvants have been shown to induce increasing magnitudes of IFN-γ responses in mice (CAF01<CAF04<CAF05) [21, 23, 24]. H56 in IC31^®^ was included as a positive control due to previously demonstrated efficacy in this model [17]. We here study a) whether the three new CAF adjuvants are immunogenic in non-human primates (NHPs) for the first time and b) the importance of the adjuvant component including their ability to induce IFN-γ responses of different magnitudes on TB disease outcome in a NHP model [17].

Overall, our main conclusion was that H56 boosted BCG-induced immunity in all four adjuvants tested with CAF04/05 inducing higher IFN-γ responses compared to CAF01 and IC31^®^, and that H56 improved disease outcomes in all adjuvants tested. Furthermore, there was no correlation between the magnitude of vaccine-specific IFN-γ response in the peripheral blood and protection. These findings caution the focus on IFN-γ in the ongoing attempts to optimize novel TB vaccines and underscore the importance of the choice of adjuvant and immunological readouts in preclinical and clinical evaluations.

## Materials and Methods

### Ethics statement and study approval

All experimental manipulations and protocols were approved by the LWM Institutional Animal Care and Use Committee (IACUC). All animals used in the study were housed in facilities accredited by the Philippine Association for Laboratory Animal Science (PALAS) in accordance with standards established in the Animal Welfare Act and the Guide for the Care and Use of Laboratory Animals.

### Experimental animals

Cynomolgus macaques (*Macaca fascicularis*) of Philippine origin bred in captivity for laboratory use ranging in age from 1.6 to 3.7 years (sub-adult to adult) and weighing 2.3–3.6 kg were used. Prior to the studies, the macaques underwent diagnostic and clinical procedures (e.g., physical examination, CBC with differential, ESR, thoracic radiography, and TST) to rule out prior exposure to Mtb. Personal care was given daily to animals by educated animal caretakers, and the monkeys’ cages were enriched with toys to improve general animal welfare. Animals were sedated prior to procedures to minimize stress, and since the only invasive procedure was blood collection and no adverse events occurred, no analgesics or further anesthetics were required. Animals were followed closely after infection, and humane endpoints were defined as weight loss exceeding 20% body mass, or when animal welfare and behavior was significantly affected by the infection. Animals were housed at the LWM Center for Leprosy and Tuberculosis Research primate facility, at Eversley Childs Compound, Jagobiao, Mandaue City, Cebu, Philippines. Individual stainless steel cages contained bars, a resting shelf and a wire mesh floor allowing monkeys to have visual, auditory, olfactory, but not physical, contact with other animals, and the monkeys received standard primate feed and fresh fruit on a daily basis and had ad libitum access to water. Due to the BCG-scab and later Mtb infection and risk of monkeys scratching each other and/or transmission of mycobacteria between monkeys, animals were single housed, however in rooms with ~10 animals per room with social but not physical interaction as mentioned. Cage floor areas were 0.4 m^2^ and height allowed normal posture and behavior. Sliding “false backs” were used to fixate animals prior to injections. The facilities windows admits natural light and as such the animals were roughly on a 12/12 hour day/night cyclus due to the proximity to equator. Fluorescent lighting was turned on at 8 AM and off at 4.15 PM daily. Animals were euthanized with an i.v. overdose of sodium pentobarbital.

### Experimental vaccines

50 μg fusion protein H56 (Ag85B—ESAT-6–Rv2660) [15] was mixed with Valneva Austria GmbH’s proprietary IC31^®^ as previously described [17]. For CAF01, CAF04 and CAF05, 50 μg of H56 was diluted in a 1:1 mixture of 10 mM TRIS-HCL buffer with pH = 7.4 and adjuvant for a total of 500 μl/dose. CAF01/04/05 were prepared as described previously [18–22]. One dose consisted of 625 μg dimethyldioctadecylammonium (DDA) bromide with 125 μg trehalose-6,6’-dibehenate (TDB: CAF01), 125 μg of the synthetic monomycoloyl glycerol analogue-1 (MMG-1: CAF04 [20]), or 125 μg TDB and 125 μg polyinosinic-polycytidylic acid (poly(I:C): CAF05) [21].

### Immunization

The study was performed at Leonard Wood Memorial Hospital (LWM), Cebu, Philippines- Five groups of n = 6 monkeys were primed with 0.1 ml BCG Danish strain (commercially available human vaccine from Statens Serum Institut, Denmark, prepared according to manufacturers instructions) intradermally (i.d.) and a sixth group was left unimmunized (n = 5, non-vaccinated). Thereafter, groups that received BCG were vaccinated intramuscularly (i.m.) in the gluteal muscle twice with 50 μg H56 in the relevant adjuvant (BCG-boost groups) as described above, or given saline (BCG only) 13 and 16 weeks after BCG. At immunizations monkeys were sedated by a cocktail of Zolitel 50 + Atrosite + Zylazil-100 mixed 5:1:1 given i.m. at 0.1 ml/kg.

### Animal infection and clinical assessments after infection

Monkeys were inoculated intratracheally six weeks after the final H56-booster (week 22) under sedation (as described above) by exposing them to a one ml solution per monkey containing 500–1000 CFU of Mtb Erdman strain. The infection was allowed to proceed until the macaques reached disease states that ranged from no apparent disease to advanced disease for a total of 71 weeks based on survival from naïve controls and BCG-boosted monkeys from previous studies also performed at LWM [7, 17]. Clinical parameters measured included weight, temperature, physical activity, appetite, complete blood counts (CBC) with differentials, chest radiographs and Sediplast Westergren erythrocyte sedimentation rate (ESR), recorded at the time points indicated in graphs. Chest radiographs were evaluated by a board-certified thoracic radiologist with extensive experience in pulmonary TB that was blinded with regards to vaccination groups. A range of different X-ray changes emerged during the study, including lateral and bilateral bronchopneumonia and pneumonia, infiltrates in hilar regions, atelectasis, calcified micro nodules, necrotizing pulmonary cavitations, and nodular densities. Some non-TB-related infiltrates and micronodules were discarded from the CXR-curves. Monkeys were euthanized when they reached one or more of a set of predetermined end points including 20% weight loss from maximum weight, loss of appetite, loss of physical activity, diarrhea or other symptoms related to TB. No monkeys were euthanized due to conditions not related to TB. In the end, euthanasia was determined by the responsible veterinarian regardless of vaccine group.

### Whole blood immune assays (proliferation and IFN-γ ELISA)

Heparinized blood (25U/ml) was diluted 1:5 with complete RPMI and stimulated with 5 μg /ml of ESAT-6 or H56 as described previously [17]. Briefly, culture supernatants were harvested after 3 days of culture and immediately replaced with ^3^H-thymidine. Proliferation was evaluated by the amount of radioactive ^3^H-thymidine incorporation after overnight stimulation. IFN-γ sandwich ELISA on whole-blood culture supernatants was performed using the monkey IFN-γ ELISA kit from U-CyTech biosciences (Utrecht, The Netherlands) on supernatants harvested as described above and following the manufacturer´s instructions.

### ELISPOT

IFN-γ ELISPOT was performed as previously described [25]. Briefly, PBMCs were stimulated with 5 μg/ml of H56 or media and incubated in ELISPOT plates coated with anti-IFN-γ antibodies for ~40 h at 37°C with 5% CO_2_ before development. Results are shown with the media background deducted. Negative responses were set arbitrarily to 0.

### Necropsy and pathology

The gross extent of pathology at necropsy from mycobacterial infection was recorded as previously described [26]. We examined for the number and size of visible granulomas for right and left lung, spleen, liver, kidney, thoracic lymph node, and other sites (pericardium, stomach, bone etc.). Scoring took into account both the number (i.e., score 0, no visible granulomas; score 1, 1–3 granulomas; score 2, 4–10 granulomas; score 3, more than 10 granulomas; score 4, miliary pattern with numerous lesions) and size (i.e., score 1, 1–2 mm; score 2, 3–4 mm: score 3, >4 mm). Each organ/entity (with pathological changes) could obtain a minimal score of 2, e.g., 1–3 small lesions (1–2 mm), and a maximum score of 7, e.g. miliary TB with several coalescent large granulomas. Note that right lungs were scored as 4 separate entities (upper, middle, lower and accessory lobes) and left lung as 3 separate entities (upper, middle, lower lobes) each with a possible maximum score of 7.

### Statistics

Pre-challenge H56 responses (IFN-γ ELISPOT, ELISA and proliferation) were compared to baseline within groups by one-way non-parametric repeated measures ANOVA (Friedmann test) and Dunn’s post-test. Responses and pathology scores between vaccine and control groups were compared by non-parametric ANOVA (Kruskall-Wallis) and Dunn’s post-test. Kaplan-Meier survival curves were compared with log-rank test (Mantel-Cox, Prism software). The Bonferroni-corrected threshold for 5 multiple comparisons (p = 0.01) was used. Weight gains (pre-challenge weight set at 100%), ESR-values, and ESAT-6 responses followed normal distributions and were compared by two-way repeated measures MANOVA with Bonferroni correction.

Correlations between different clinical parameters as well as between clinical parameters and vaccine responses were performed using Pearson’s product-moment correlation coefficient (R) and correlation test, except time-to-death values. Since these values were clearly not normally distributed around the correlation line, non-parametric Spearman’s product moment test was used instead. GraphPad prism 5.0 software was used for analysis.

### Combined analysis of three BCG-H56/IC31^®^ experiments in cynomolgus macaques

A combined statistical analysis that included data from two previously published experiments of BCG boosted with H56 in IC31^®^ [17] was performed. One of the previously published experiments, named experiment 2, was performed at the same facility as experiment 1 (the current study), and had the same overall timing in vaccination and challenge, as well as the same challenge dose. A third experiment was conducted at the primate center at the University of Pittsburgh with a lower challenge dose, experiment 3, of 25 CFUs per monkey. The timing of vaccinations in experiment 3 was as follows: BCG was given at week 0, H56/IC31^®^ boosters were given at week 8 and 12. Challenge was given at week 20. The three experiments all contained the same three treatment groups, non-vaccinated, BCG and BCG boosted with H56 in IC31^®^ (BCG-H56/IC31). To account for the intrinsic differences between these experiments a stratified Cox regression analysis was performed, in which each of the three experiments were allowed to have their own underlying hazard function, showing no significant difference between vaccine effect between the experiments (p = 0.56, see supporting information). This analysis was performed in SAS v9.3 (SAS Institute, Cary, NC).

## Results

**Fig 1:**
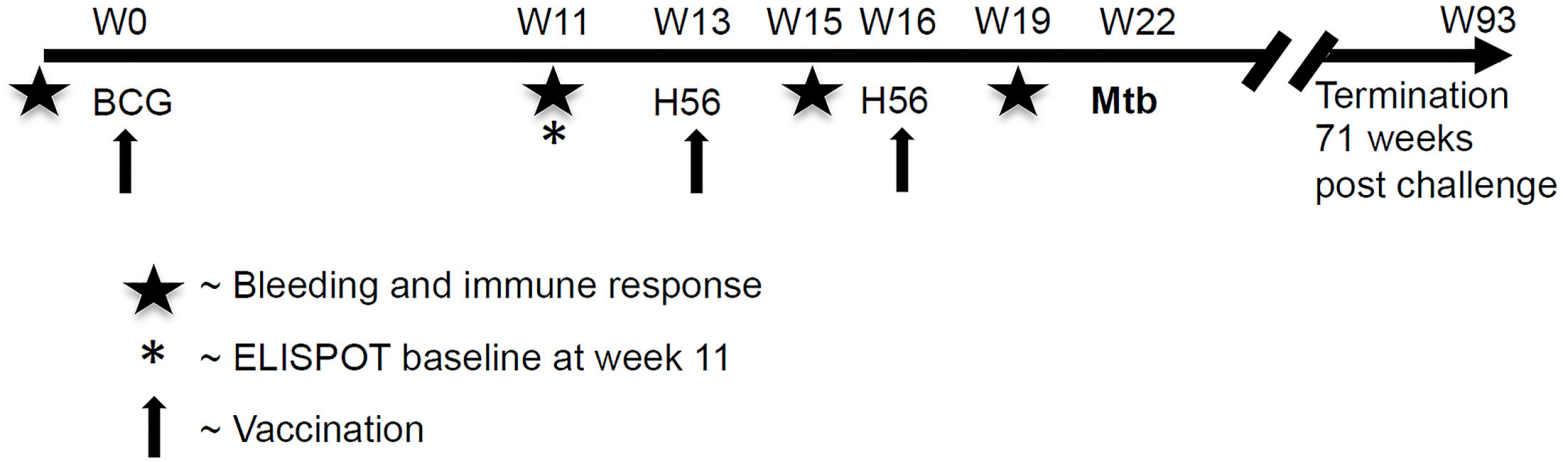
Schematic overview of the experimental time line for NHP immunization, Mtb challenge, necropsy and sampling. Clinical parameters including weight, ESR, CXR was recorded at regular time points after challenge as indicated in graphs. See MM for details.

We assessed immunogenicity and efficacy of boosting BCG with H56 in four different adjuvants, CAF01 (DDA/TDB), CAF04 (DDA/MMG), CAF05 (DDA/TDB/polyI:C), and IC31^®^. Monkeys (n = 6/group) were given BCG Danish i.d. followed by H56 i.m. in the different adjuvants (or given saline only for the BCG only group), 13 and 16 weeks later (see Fig 1 for experimental setup). The control group (non-vaccinated, n = 5) was left unimmunized. Monkeys were infected 6 weeks after the last H56-booster by the intratracheal route with an intermediate dose [27, 28], and monitored closely for clinical parameters (including weight, ESR, CXR) for a period of 71 weeks after challenge based on previous studies [7, 17], and necropsied when reaching predefined euthanasia criteria (see MM).

### H56 in four different cationic adjuvant formulations promote different levels of IFN-γ responses

**Fig 2:**
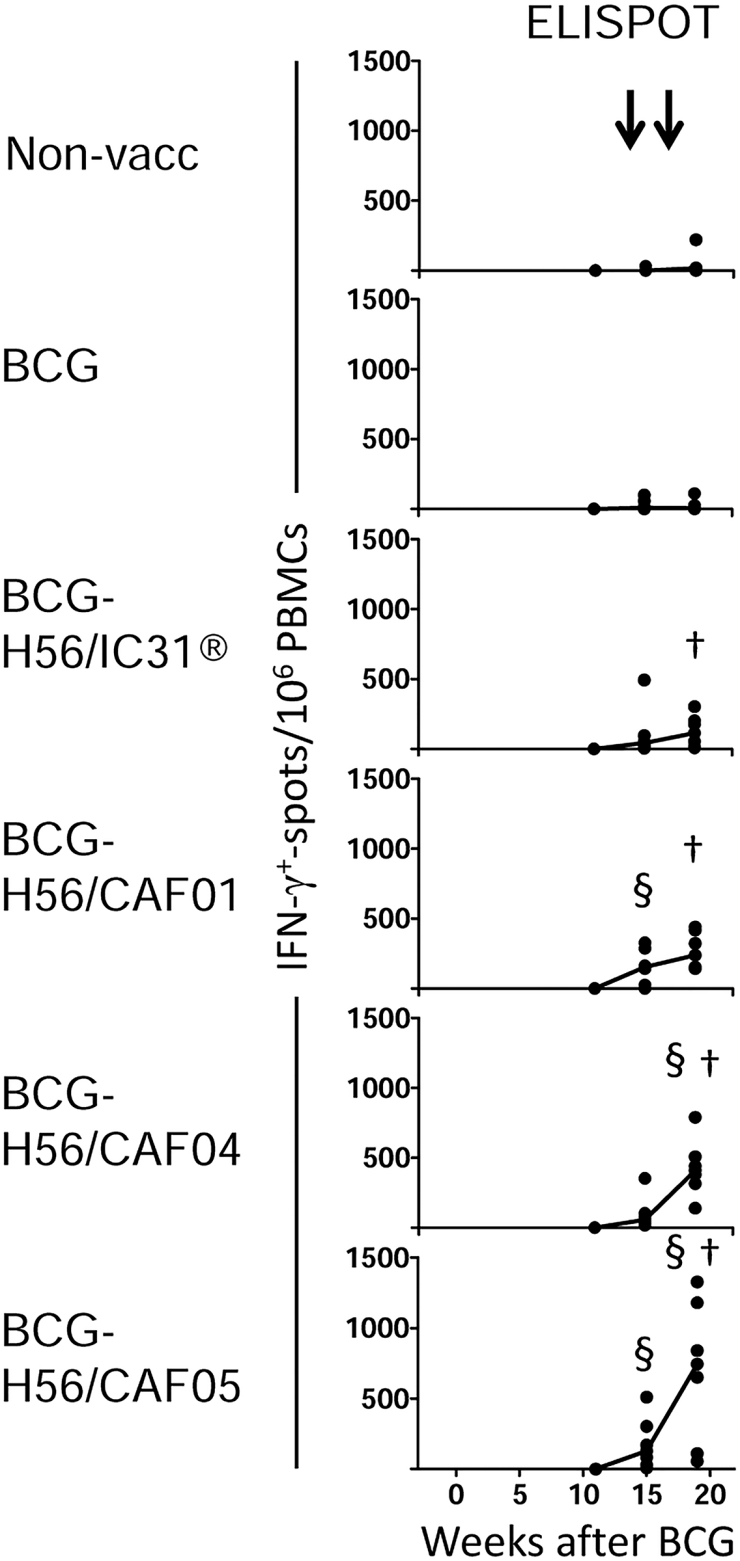
Boosting BCG with H56 in four cationic adjuvants induces vaccine-specific proliferation and IFN-γ production. Monkeys were immunized and bled as depicted in Fig 1, timing of BCG and H56 boosters are indicated with arrows above the panels. The number of IFN-γ producing cells in PBMCs assessed by ELISPOT after H56 stimulation *in vitro* at the indicated time points in duplicate cultures. Note ELISPOT baseline was at week -11. Dots depict background-subtracted responses of individual monkeys; medians are indicated with a solid line. Medians for non-vaccinated and BCG groups were zero. †, denotes significant difference compared to baseline within group. §, denotes significant difference compared to non-vaccinated at the same time point. See text for p-values.

Vaccine-specific immune responses in peripheral blood after H56-boosters were monitored by IFN-γ ELISPOT. All H56-boosted groups elicited significant H56-specific IFN-γ ELISPOT responses after the second H56 boost compared to the baseline response within the same group (Fig 2). The highest IFN-γ values were obtained in the BCG-H56/CAF05 group (median spots/10^6^ PBMCs +/- interquartile range, 745 +/- 630, p<0.01 compared to baseline) followed by BCG-H56/CAF04 (410 +/-126.3, p<0.01). BCG boosted with H56 in CAF01 and IC31^®^ both resulted in more modest responses (237.5 +/-218.8, p<0.01 and 113.8 +/-155, p<0.05, respectively), and except for the BCG-H56/IC31^®^ group, these responses reached statistical significance when compared to naïve controls (p<0.05; Fig 2). Immune responses after H56 boosts were also monitored by proliferation and IFN-γ ELISA, and H56-responses of all monkeys (n = 35) were ranked from 1 (lowest response) to 35 (highest response) separately for each of the three immune readouts, IFN-γ ELISPOT, IFN-γ ELISA, and proliferation. The sum of the ranked values supported that the adjuvants induced high (CAF04 and CAF05) or low (CAF01 and IC31^®^) responses differing significantly from each other (p<0.05) and also from the control groups (p<0.05–0.001; S1 Fig).

### Survival

**Fig 3:**
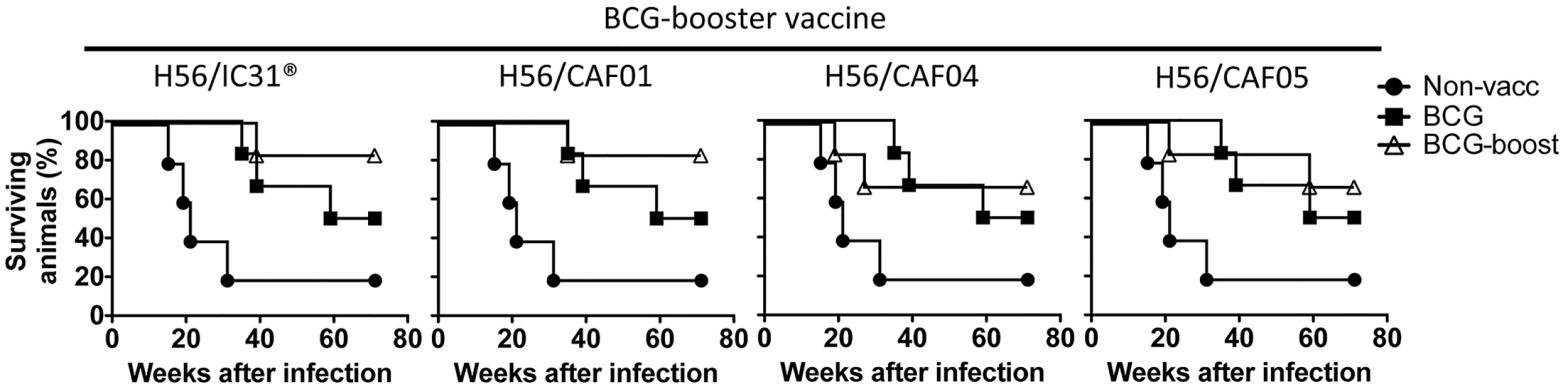
Boosting BCG with H56 improves survival after Mtb infection. Kaplan-Meier survival curves of the different BCG-booster vaccine groups (open triangles) are shown against the non-vaccinated group (filled circles) and BCG only group (filled squares). Symbols indicate euthanasia events.

All monkeys were challenged with Mtb by the intratracheal route six weeks after the second H56-boost, and development of clinical TB followed for up to 71 weeks based on previous studies [7, 17]. Infection with 500–1000 CFU Mtb resulted in lethal pulmonary TB in 4/5 uninfected monkeys in line with previous studies [17, 27]. Animals were euthanized based on predefined criteria (see MM). All BCG-H56 boosted groups had better survival rates (BCG-H56/IC31^®^ and BCG-H56/CAF01; 5/6 survivors (83% survival), BCG-H56/CAF04 and BCG-H56/CAF05; 4/6 survivors or 67% survival) compared to BCG alone (3/6 survivors or 50% survival) and non-vaccinated (1/5 survivors (20% survival), Fig 3). However, none of the H56-boosted groups significantly prolonged median survival time compared to BCG alone. We then compared Kaplan-Meier survival curves of all groups by a log-rank test to also take timing of deaths into account and not only median survival times. The initial log-rank test of all the groups showed a significant overall effect of vaccination (p = 0.03), indicating that survival curves of the groups differed. However, due to the small sample sizes (6 monkeys per vaccine group and 5 non-vaccinated controls), these differences were not statistically significant when survival curves of individual groups were analyzed against each other and corrected for multiple comparisons. The survival curve and median survival time of all the BCG-H56 boosted monkeys combined as one group was significantly different compared to the non-vaccinated group (p<0.01), but not to the BCG-only group.

### Post challenge assessment of clinical parameters and immune responses

**Fig 4:**
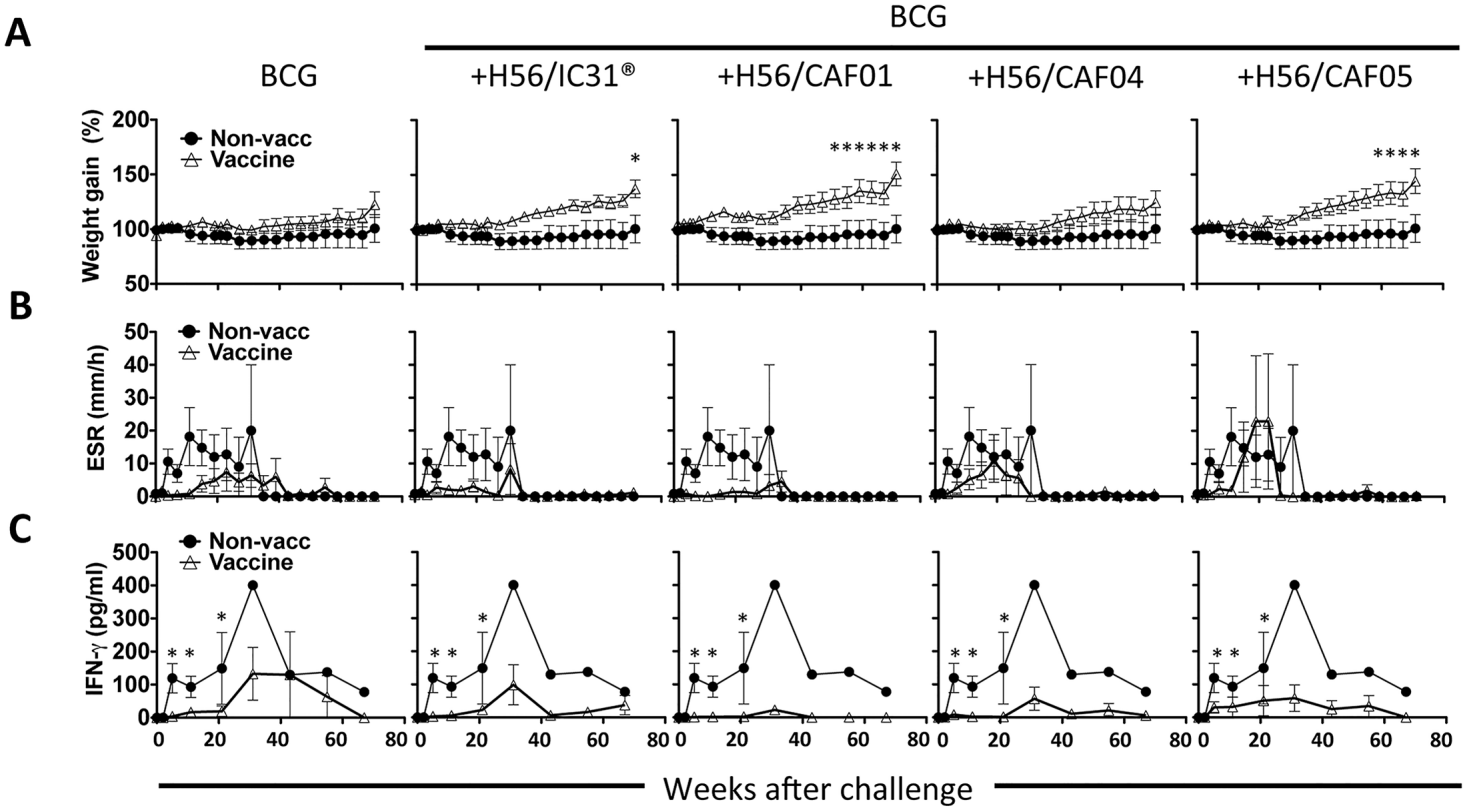
Boosting BCG with H56 improves clinical parameters and dampens infection-driven immune responses after Mtb infection. A, weight gains post challenge. Lines represent mean +/- SEM of relative weight gains. Weight at the time of challenge was set to 100% for each animal. *, denotes statistical significance when compared to the non-vaccinated group. The non-vaccinated group is depicted by filled circles in all panels against each vaccine group (open triangles). B, erythrocyte sedimentation rate (ESR) post challenge. Lines represent mean +/- SEM values post challenge for each group. For monkeys necropsied prior to the study termination, their weights and ESR-values at the necropsy time point were used for the remainder of the study. Symbols as described in A. C, post challenge ESAT-6 specific responses were assessed by IFN-γ ELISA. Lines represent mean +/- SEM IFN-γ production of triplicate whole blood cultures stimulated with ESAT-6, with background (media control) deducted. It should be noted that only one non-vaccinated monkey survived after week 27, hence the lack of error bars in C after this time point. *, denotes statistical significance when compared to the non-vaccinated group. Comparisons were done for weeks 0–21 only since later time points only included one animal in the non-vaccinated group. Symbols as described in A.

All vaccinated monkeys gained more weight during the course of infection compared to the non-vaccinated controls, and the highest weight gains were observed in the BCG-H56/CAF01 group followed by BCG-H56/CAF05 and BCG-H56/IC31^®^ groups, which were significantly different from non-vaccinated at the time points indicated by asterisks in Fig 4A (p<0.05–0.001). No other groups were found to differ significantly.

ESR was used as a measurement of general inflammation during the course of infection. The results showed that all groups receiving BCG had lower average ESR-readings after challenge compared with non-vaccinated monkeys except the BCG-H56/CAF05 group (Fig 4B), and further, that the lowest ESR-levels were observed in the groups with the highest survival rates (BCG-H56 in CAF01 and IC31^®^).

Non-vaccinated monkeys developed high T cell responses against ESAT-6 during the first five weeks of infection, which lasted throughout the study as measured by IFN-γ ELISA (Fig 4C). In contrast, the BCG only group had a delayed onset and decreased magnitude of ESAT-6 response, which was even more apparent in the BCG-H56 boosted groups (Fig 4C). ESAT-6 responses were significantly higher in non-vaccinated compared to the remaining groups, but no other groups differed significantly. The H56 response post infection closely resembled the ESAT-6 response (not shown).

During the course of infection, monkeys were regularly inspected for changes in CXR radiography, resulting in a range of different X-ray changes (see MM). All immunized groups showed a delayed development of X-ray changes as well as fewer X-ray changes compared to the non-vaccinated group although this was not statistically significant (S2 Fig).

### Post challenge assessment of pathology

**Fig 5:**
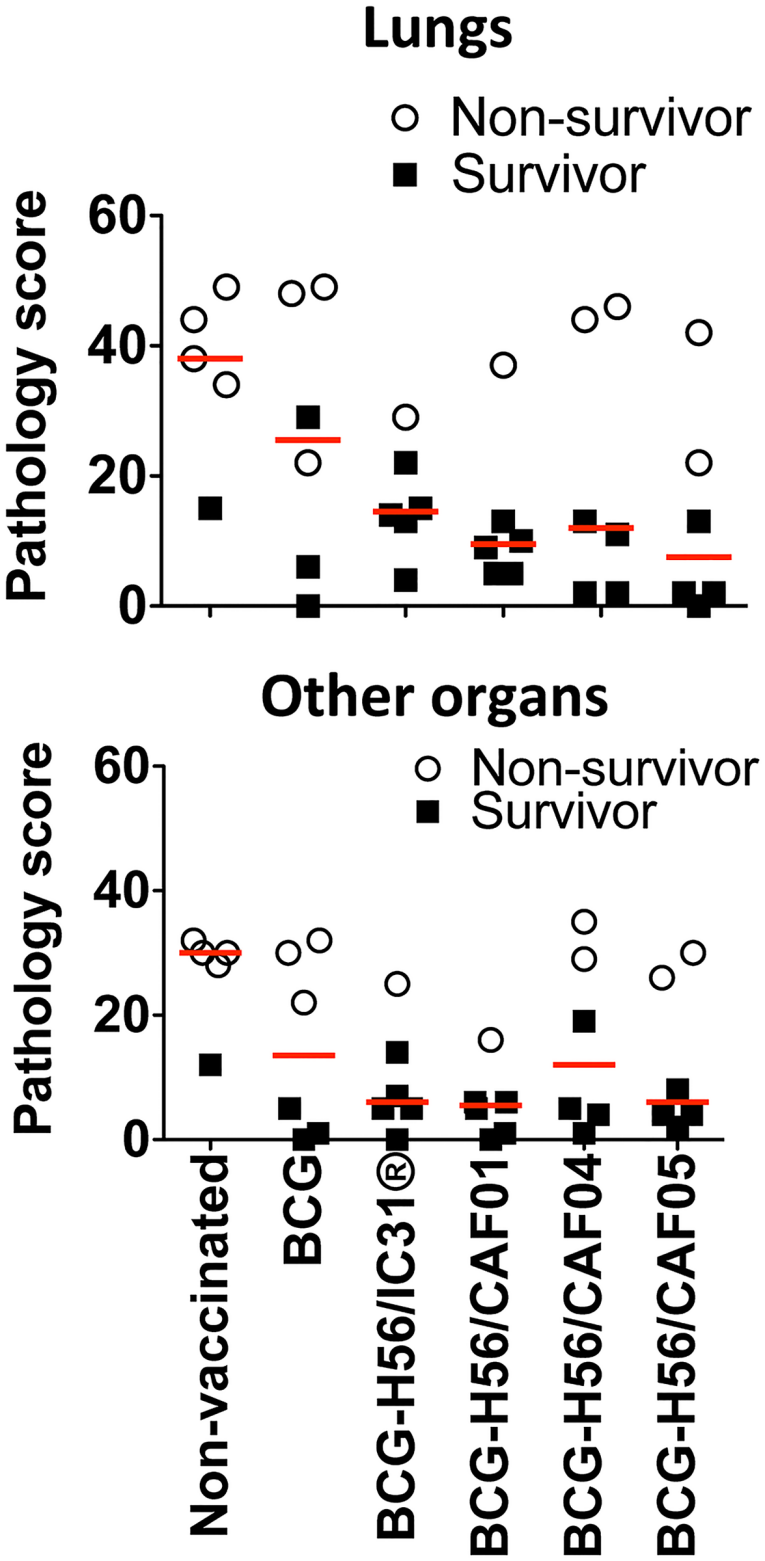
Lung and disseminated pathology in Mtb infected NHPs after boosting BCG with H56. Pathology scores in lungs and other organs (arbitrary units, for pathology scoring see MM) are shown for individual monkeys within different experimental vaccine groups. Median pathology scores are indicated with red horizontal lines for each group. Non-survivors, open circles, survivors, solid squares.

TB-related pathology was evaluated by the method previously described by Flynn and colleagues [26] (see MM). Monkeys developed a range of pathological manifestations of TB in lungs and other organs including miliary TB, tuberculous pneumonia, consolidation, congestion, as well as granulomas ranging from small discrete to large and coalesced with extensive eosinophilic and neutrophilic necrosis and liquefaction (Fig 5). Non-surviving animals clearly had more TB-related pathology than survivors regardless of vaccine group (p<0.0001; not shown), and the trend was that groups boosted with H56 displayed the lowest median pathology scores in lungs other organs (Fig 5). Only in the BCG-H56/CAF01 group did the differences between the non-vaccinated control group and vaccine group reach statistical significance (with less extra-pulmonary lesions; median with 25% percentiles, 0.5, 0–4.25) compared to non-vaccinated controls (15, 10–17; p<0.05, not shown).

### Correlations of clinical measurements and vaccine responses to challenge outcome

**Fig 6:**
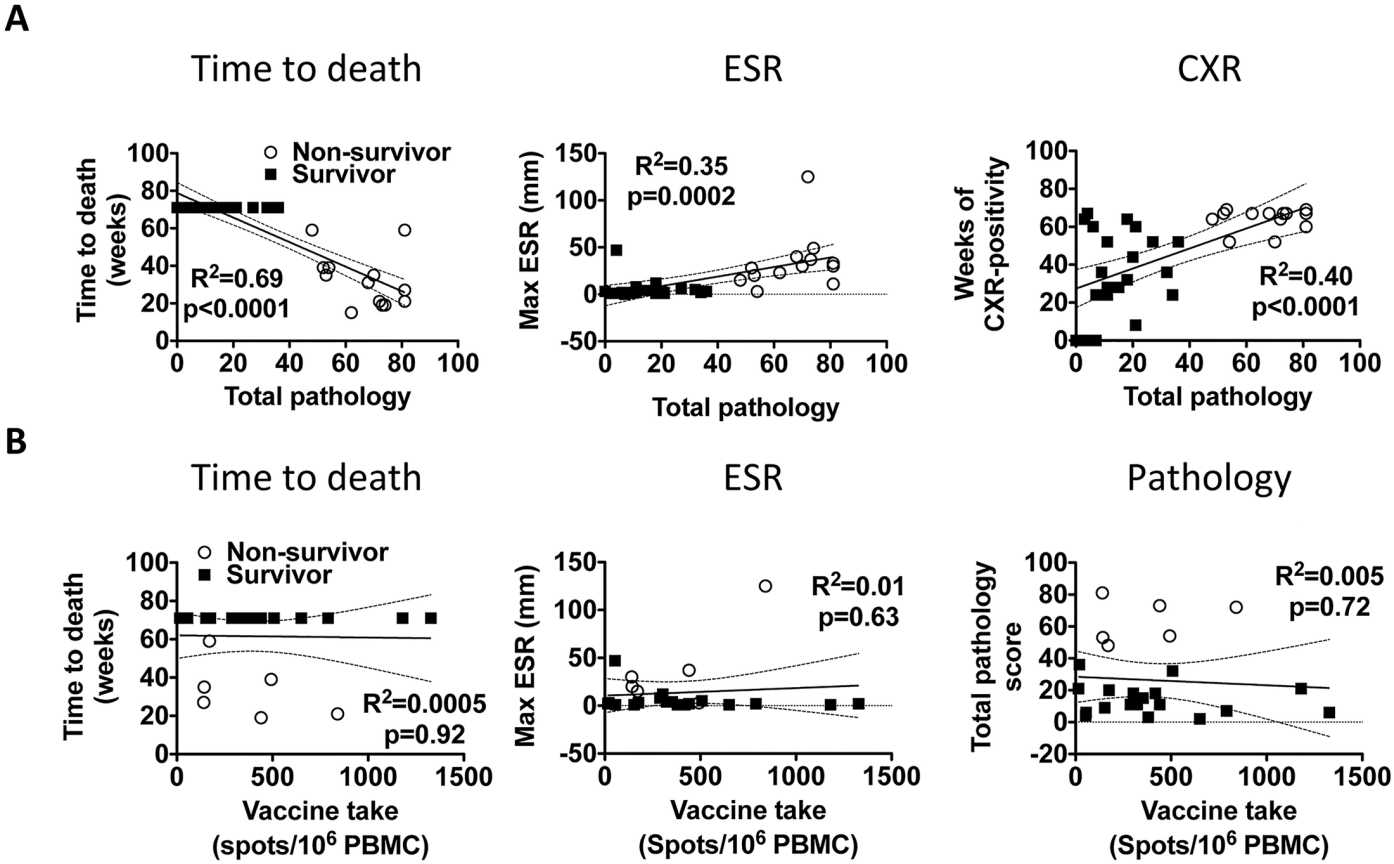
Significant correlation between clinical parameters but not between H56-vaccine promoted IFN-γ production and protection. A, the clinical parameters: 1) time to death, 2) the maximum ESR-value post challenge, and 3) weeks of chest X-ray (CXR) positivity after challenge are plotted against the total level of pathology (summed lung and extra-pulmonary) for all individual monkeys (n = 35). B, the clinical parameters: 1) time to death, 2) maximum ESR-reading post challenge and 3) total pathology scores are plotted against the maximum H56 response measured by ELISPOT pre-challenge for individual monkeys in the H56 booster groups (n = 24). A and B, correlations between different clinical parameters as well as between clinical parameters and vaccine responses were performed using Pearson’s product-moment correlation coefficient (R) and correlation test for ESR, CXR, and pathology, and the non-parametric Spearman’s product moment test for time to death. The coefficient of determination (R^2^) with 95% CI (dotted lines) are shown.

We next performed linear regression between the different clinical parameters in order to validate if they reflected TB disease burden. We also performed correlations between vaccine responses and clinical parameters to search for correlates of protection. All the clinical parameters tested (time to death, ESR, CXR) correlated closely with the level of gross pathology, underscoring their validity to reflect the disease state of the corresponding animal (Fig 6A). However, the magnitude of H56-vaccine response in H56-boosted animals as measured by IFN-γ ELISPOT pre-challenge (or proliferation and IFN-γ release measured by ELISA; not shown), did not correlate with time to death, ESR-levels, pathology scores (Fig 6B), or CXR changes (not shown). H56 vaccine responses in H56-boosted animals did not correlate with weight gains or post challenge responses against H56 or ESAT-6 either (not shown). Interestingly, peak H56 post challenge responses were significantly increased in non-survivors compared to survivors, and correlated positively with pathology scores (p<0.001 and p<0.0001, respectively; S3 Fig). This clearly indicated that even under controlled experimental circumstances there was no straightforward relationship between the level of IFN-γ specific to the vaccine antigen, H56, and protection against TB.

## Discussion

This is the first study in the NHP TB model to compare the efficacy of the same vaccine antigen administered in different adjuvants that promote IFN-γ production of different intensities and correlate the vaccine IFN-γ responses with long-term survival.

The study’s main conclusion was that boosting BCG with H56 in 4 different adjuvants all improved protection over BCG alone, albeit at non-significant levels. The trend was that H56 given in CAF01 and IC31^®^ promoted the best protection. Secondly, we found a strong correlation between the different clinical parameters used to assess TB severity, but no correlation between the clinical parameters and the magnitude of vaccine IFN-γ responses, at neither the group (not shown) nor individual monkey level (Fig 6). Interestingly, H56 in the different adjuvants induced a hierarchy of response highly similar to that observed in mice [21, 23, 24]. Non-protected animals (non-survivors) had higher post-challenge peak H56 (and ESAT-6, which is included in H56) responses compared to survivors and furthermore post challenge ESAT-6/H56 peak responses correlated directly with disease severity (Fig 4 and S3 Fig). Most monkeys also developed high responses to ESAT-6 and H56 shortly before reaching the predefined euthanasia endpoints (not shown). This demonstrates that systemic ESAT-6 (and H56) responses post challenge reflected Mtb-induced responses rather than vaccine responses, and that the H56 vaccine seemed to counteract infection through mechanisms distinct from a systemic IFN-γ recall response upon challenge. Alternatively, the responses measured in the peripheral blood in the current study did not reflect pulmonary responses at the site of infection, as suggested by a recent study [29]. The recent advances in understanding the protective capacity of CXCR3^hi^/KLRG-1^lo^ CD4 T cells located in the lung parenchyma of mice versus CXCR3^lo^/KLRG-1^hi^ CD4 T cells located in the vasculature instead expressing CX3CR1 would also argue that even if circulatory IFN-γ producing T cells did not correlate with protection, local pulmonary T cells might have [6].

The lack of correlation between vaccine-promoted IFN-γ responses in peripheral blood and protection extend previous observations from TB vaccination studies in the mouse model [5–7], and is also in agreement with the lack of correlation between IFN-γ (or any other immunological read-out measured) and protection in BCG vaccinated infants [8]. Importantly, this is in contrast with studies in mice and cattle showing direct correlations between IFN-γ production and protection [9–11] and is also contrary to the study of Verreck and colleagues who found that vaccine-specific IFN-γ release correlated with protection after boosting BCG with MVA85A in NHPs [12]. However, the Verreck study used short-term pathology 17 weeks post challenge as the protective readout and it was performed in rhesus macaques (RMs) in contrast to the cynomolgus macaques we used in the current study, potentially explaining the conflicting results on correlation between IFN-γ release and protection in the two studies. Furthermore, the protection observed by BCG-MVA85A was not recapitulated in a long-term survival NHP study also using RMs [30]. It is important to keep in mind that the main goal of this study was to compare immunogenicity and protection of H56 in different adjuvants rather than approving or disapproving the correlative value of systemic IFN-γ production.

The initial phase IIb efficacy trials of BCG boosting with MVA85A gave very disappointing results with no increased protection compared to BCG alone. Very limited NHP data are available for this vaccine and whereas this vaccine has shown some protection measured as pathological changes 17 weeks after challenge in a RM model of TB [12], it failed in a long term RM survival study [30], which so far recapitulates findings in humans. The only vaccine to date to have been tested in both cynomolgus macaques and RM is BCG, and some evidence suggest better protection in cynomolgus macaques [31]. It will therefore be important to determine which monkey TB model, RM or cynomolgus macaques, if any, is the better predictor of human anti TB vaccine efficacy. The RM TB model is characterized by a more aggressive infection leading to acute disease in most cases, whereas the low dose cynomolgus macaque model as developed by Joanne Flynn´s laboratory results in a more protracted infection and up to 50% latent infection, with the latter resembling human infection with Mtb more closely [31, 32]. However, more clinical efficacy data from other vaccine candidates that have been tested for protection against TB in RM and/or cynomolgus macaques are needed to conclude on the most predictive model.

Bacterial loads in terms of lung CFUs would obviously have been a relevant parameter in our study, however, a reliable protocol for sampling granulomas for plating and counting lung CFUs had not been developed at the study site when this experiment was carried out. As a result, a veterinarian who was blinded with regards to vaccination groups dissected representative lung tissue for plating at necropsy for each animal, but these samples harbored very few culturable bacteria yielding highly variable and unreliable results that we were not confident in including.

It has been previously shown by Overwijk and colleagues in cancer research that immunization in emulsion adjuvants such as IFA induces sequestration, retention and even deletion of vaccine specific T cells at the antigen-containing vaccination site, hindering T cells in infiltrating and combating the tumor and a resulting failure of such vaccines [33]. As CAF adjuvants also create a depot at the injection site, a sequestration effect cannot be completely ruled out in our study, however, antigens emulsified in CAF adjuvants are cleared from the injection site “depot” in roughly 14 days which would be well before the monkeys were challenged with Mtb. [34], and CAF adjuvants create less local inflammation compared to IFA. Furthermore, strong retention/sequestration of vaccine specific T cells at the injection site after H56 booster vaccinations could have caused a visible delayed type 1 hypersensitivity (DTH) reaction at the injections site, which was not the case. However, Overwijk and colleagues found that sequestered T cells underwent apoptosis at the injection site, and in this case a DTH reaction would not necessarily have been expected. In mice models of tuberculosis, we observe long-lived memory T cells after immunization with vaccines in CAF adjuvants, and these cells survive and retain their proliferative potential and memory phenotype systemically and in the lungs months after infection [7, 35]. Hence, not all vaccine-specific T cells are sequestered/deleted at the injection site after infection. Finally, in the past decade of extensive vaccine testing in different animal models, significant sequestration/deletion of vaccine specific T cells at the injection site has not been observed in histopathological samples from toxicology/safety examinations of the injection site after CAF immunizations, nevertheless, the potential negative impact of sequestration/deletion of vaccine specific T cells at the injection site deserves further examination.

A number of non-Th1 related immune functions may play an important role in immunity against Mtb [36–38], and although our study was not designed to define the mechanism of protection, it is relevant to note that the H56 subunit vaccines adjuvanted with CAF01 and IC31^®^ (both of which induced the highest level of protection in the face of very modest levels of IFN-γ) previously has been reported to promote a response dominated by central memory-like T cells (Tcm) that produce TNF-α and IL-2 but not IFN-γ [39, 40]. Tcm’s are critically important for maintaining long-term memory after vaccination and crucial for sustained control with TB infection [7, 35, 41]. The fact that increasing strength of the IFN-γ signal in the H56-vaccinated groups had no beneficial influence on protection against Mtb infection (in fact the tendency was the opposite), may in this context suggest that the “stronger” adjuvants (CAF04/05) changed this balance to a more effector-like T cell response, however, this is currently under investigation. This would be in line with a previous study from our lab in which boosting a related adjuvanted fusion protein (H28, based on TB10.4 instead of ESAT-6) with MVA expressing H28 increased the response and changed it into a more effector-driven response, but resulted in less protection compared to adjuvanted H28 alone in mice and NHPs [7]. A similar shift of balance towards more effector-like T cell responses has been observed in previous studies when comparing “stronger” viral infections with high viral loads to “weaker” infections with lower viral loads (reviewed in [42]). Unfortunately, no detailed T cell phenotyping was performed in the present study to address these issues due to technical limitations at the study site in the Philippines and loss of viability in shipped cryopreserved PBMCs preventing multicolor flow cytometric analysis. Such in-depth analyses along with unbiased whole-genome systems biology approaches will be essential in future studies in order to enhance our understanding of a protective anti-TB immune response and immune correlates of protection. However, such analysis will require robust protection against TB over BCG alone, and larger groups sizes (n>20) will be essential in such studies to obtain the sufficient statistical power to reach firm conclusions.

Boosting BCG with H56/IC31^®^ has now been tested in three independent experiments (the current study and two previously published experiments [17], all of which led to an improvement, albeit non-significant, of BCG-induced immunity and protection. An analysis of the combined survival curves of these three experiments provided statistical power for a more robust analysis resulting in statistically significant (p = 0.05) log-rank tests between survival curves of non-vaccinated, BCG, and BCG-H56/IC31^®^ by one-sided and borderline significance (p<0.07) for two-sided analyses (see S4 Fig). Importantly, the overall effect of vaccination of the log-rank test (without post-test single comparisons) was p = 0.004, indicating groups were indeed different. This clearly demonstrates the need to increase group sizes in ongoing attempts to evaluate novel vaccines in NHP models when the endpoint is survival. However, recent breakthroughs in the application of PET-CT for the *in vivo* monitoring of ongoing TB infection and disease in NHP´s [43], may enable accelerated testing and smaller group sizes in future vaccine studies. Eventually large-scale human efficacy trials of vaccines that were protective in the NHP model are needed to validate the predictive value of this model for protection of these vaccines in humans—a predictive value that as of yet stands unconfirmed despite this model’s close resemblance to human TB. This would allow a rational progression towards stage gating and prioritization of vaccines for future clinical trials.

## Supporting Information

S1 Fig Immunogenic hierarchy of H56 in 4 adjuvants in NHPs. Three weeks after the second H56 booster vaccination, monkeys were bled and immune responses were assessed by ELISPOT on PBMCs (A), as well as proliferation (B) and ELISA (C) on whole blood (see MM for details). Samples were stimulated with H56 *in vitro* and background from media controls were deducted from ELISA and ELISPOT values. Dots depict responses of individual monkeys; medians are indicated with a solid line. Groups were compared Kruskall-Wallis non-parametric test with Dunn’s post test for multiple comparisons. *, p<0.05, **, p<0.01. Responses were compared to baseline (not shown) by Friedmann test and Dunn’s post test for multiple comparisons. #, p<0.05, §, p<0.01. D, responses for all individual monkeys (n = 35) were ranked from 1 (lowest response) to 35 (highest response) separately for the ELISPOT, proliferation and ELISA results shown in A-C. Each data point in D represent the sums of the ranked values (3 per monkey) for each individual monkey. Summed ranks were normally distributed and compared between all groups by one-way ANOVA with Newman-Keuls post-test for multiple comparisons of individual vaccine-groups. *, p<0.05, ***, p<0.001.
(TIF)

S2 Fig CXR changes after challenge. CXR-changes as measured by chest radiographs. The graphs depict the percentage of monkeys without chest X-ray changes. A positive slope in CXR-curves indicates resolution of CXR-changes in an individual monkey. CXR-positive monkeys that were euthanized before the study termination at week 71 were plotted as positive for the remainder of the study. CXR “survival” curves did not differ significantly by a log-rank test. The non-vaccinated group is depicted by open triangles in each panel against the indicated vaccine group (closed circles).
(TIF)

S3 Fig Peak H56 responses post challenge separate survivors from non-survivors and correlate with pathology. A, the maximum H56 response measured by IFN-γ ELISPOT post challenge for individual animals is shown regardless of vaccine group for survivors (filled squares) and non-survivors (open circles). Each symbol represents the maximum H56 response with media background values deducted post challenge for one monkey. Responses between survivors and non-survivors were compared by a Mann-Whitney test. B, the correlation between the total pathology score (summed lung- and extra-pulmonary pathology scores) and maximum H56 response measured by ELISPOT as described in A for individual monkeys is shown. Correlation was performed by Spearman’s product moment correlation test. Symbol legends as in panel A.
(TIF)

S4 Fig Boosting BCG with H56 in IC31^®^ improves survival compared to BCG alone in three independent NHP experiments. A, Kaplan-Meier survival curves of the three experiments assessing protection of BCG-H56 in IC31^®^ are shown. Only the current study (Exp. 1) also included BCG-booster groups with H56 in other adjuvants than IC31^®^. Exp. 3 was a low-dose challenge study, whereas exp. 1 and 2 were medium-high dose challenge experiments performed at the same study site (LWM). Experiments 2 and 3 were published previously [17]. See MM for details. B, summary of the stratified Cox regression analysis of experiment 1–3. With the differences between the three experiments with respect to timing in vaccination and challenge dose kept in mind, a stratified Cox regression model was selected, in which each experiment was assumed to have its own individual hazard function (risk of death per time unit). As a cautious first step of analysis we tested for identical treatment effects in the three studies, which was accepted (p = 0.56). Hence, there was no significant difference between vaccine efficacies in the three NHP experiments. The overall treatment effect on survival of the three groups was clearly significant by log-rank test (p = 0.004). Since boosting BCG with H56 has consistently improved protection compared to BCG alone in murine and NHP models (i.e. H56 boost of BCG have never resulted in impaired outcome) [15, 17], we performed individual comparisons as one-sided statistical tests, but have also provided p-values for the more conservative two-sided tests.
(TIF)
