# Supplementary figures and images for: Testing the H56 Vaccine Delivered in 4 Different Adjuvants as a BCG-Booster in a Non-Human Primate Model of Tuberculosis
Source: PLoS One. 2016 Aug 15;11(8):e0161217. doi: 10.1371/journal.pone.0161217 (PMC4985151; doi:10.1371/journal.pone.0161217)

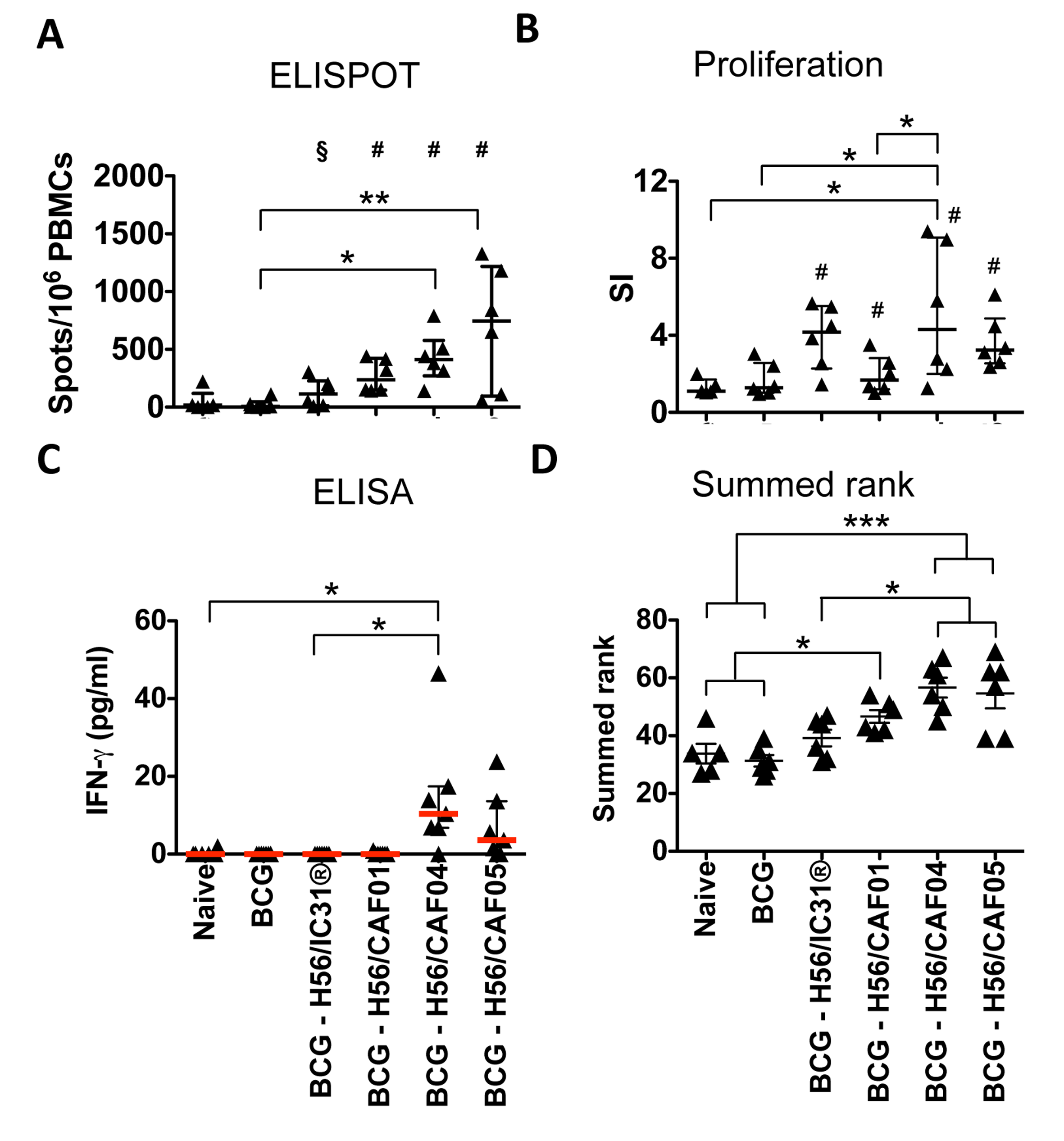

Supplement: S1 Fig — Three weeks after the second H56 booster vaccination, monkeys were bled and immune responses were assessed by ELISPOT on PBMCs (A), as well as proliferation (B) and ELISA (C) on whole blood (see MM for details). Samples were stimulated with H56 in vitro and background from media controls were deducted from ELISA and ELISPOT values. Dots depict responses of individual monkeys; medians are indicated with a solid line. Groups were compared Kruskall-Wallis non-parametric test with Dunn’s post test for multiple comparisons. *, p<0.05, **, p<0.01. Responses were compared to baseline (not shown) by Friedmann test and Dunn’s post test for multiple comparisons. #, p<0.05, §, p<0.01. D, responses for all individual monkeys (n = 35) were ranked from 1 (lowest response) to 35 (highest response) separately for the ELISPOT, proliferation and ELISA results shown in A-C. Each data point in D represent the sums of the ranked values (3 per monkey) for each individual monkey. Summed ranks were normally distributed and compared between all groups by one-way ANOVA with Newman-Keuls post-test for multiple comparisons of individual vaccine-groups. *, p<0.05, ***, p<0.001. (TIF) [file pone.0161217.s001.tif]

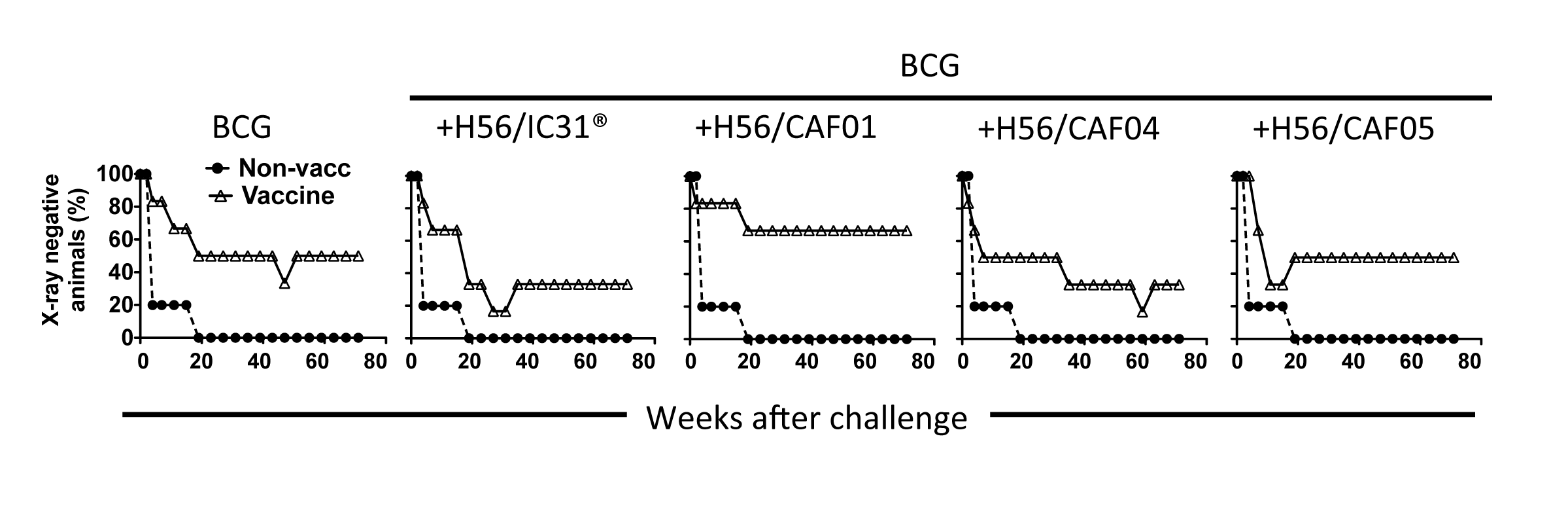

Supplement: S2 Fig — CXR-changes as measured by chest radiographs. The graphs depict the percentage of monkeys without chest X-ray changes. A positive slope in CXR-curves indicates resolution of CXR-changes in an individual monkey. CXR-positive monkeys that were euthanized before the study termination at week 71 were plotted as positive for the remainder of the study. CXR “survival” curves did not differ significantly by a log-rank test. The non-vaccinated group is depicted by open triangles in each panel against the indicated vaccine group (closed circles). (TIF) [file pone.0161217.s002.tif]

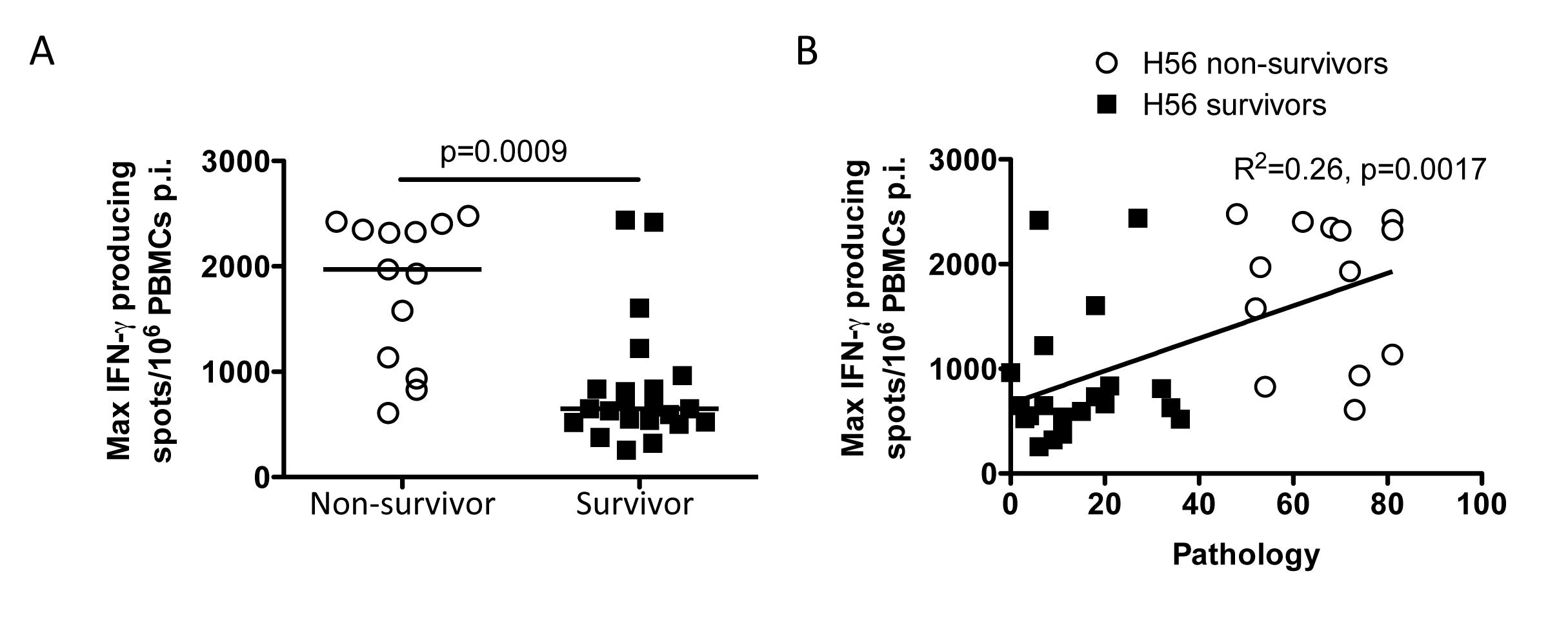

Supplement: S3 Fig — A, the maximum H56 response measured by IFN-γ ELISPOT post challenge for individual animals is shown regardless of vaccine group for survivors (filled squares) and non-survivors (open circles). Each symbol represents the maximum H56 response with media background values deducted post challenge for one monkey. Responses between survivors and non-survivors were compared by a Mann-Whitney test. B, the correlation between the total pathology score (summed lung- and extra-pulmonary pathology scores) and maximum H56 response measured by ELISPOT as described in A for individual monkeys is shown. Correlation was performed by Spearman’s product moment correlation test. Symbol legends as in panel A. (TIF) [file pone.0161217.s003.tif]

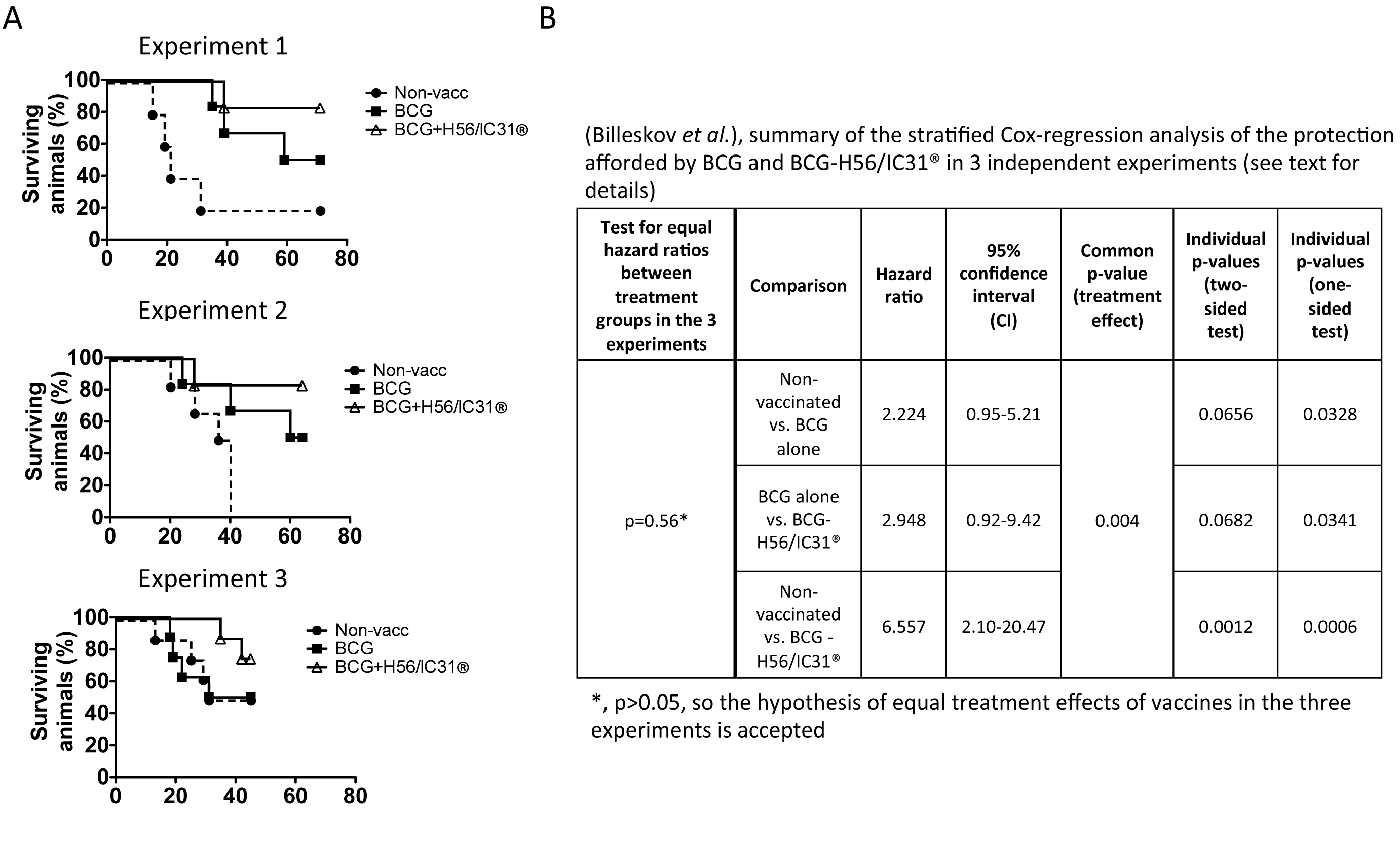

Supplement: S4 Fig — A, Kaplan-Meier survival curves of the three experiments assessing protection of BCG-H56 in IC31® are shown. Only the current study (Exp. 1) also included BCG-booster groups with H56 in other adjuvants than IC31®. Exp. 3 was a low-dose challenge study, whereas exp. 1 and 2 were medium-high dose challenge experiments performed at the same study site (LWM). Experiments 2 and 3 were published previously [17]. See MM for details. B, summary of the stratified Cox regression analysis of experiment 1–3. With the differences between the three experiments with respect to timing in vaccination and challenge dose kept in mind, a stratified Cox regression model was selected, in which each experiment was assumed to have its own individual hazard function (risk of death per time unit). As a cautious first step of analysis we tested for identical treatment effects in the three studies, which was accepted (p = 0.56). Hence, there was no significant difference between vaccine efficacies in the three NHP experiments. The overall treatment effect on survival of the three groups was clearly significant by log-rank test (p = 0.004). Since boosting BCG with H56 has consistently improved protection compared to BCG alone in murine and NHP models (i.e. H56 boost of BCG have never resulted in impaired outcome) [15, 17], we performed individual comparisons as one-sided statistical tests, but have also provided p-values for the more conservative two-sided tests. (TIF) [file pone.0161217.s004.tif]
